# The integration of the global HIV/AIDS response into universal health coverage: desirable, perhaps possible, but far from easy

**DOI:** 10.1186/s12992-019-0487-5

**Published:** 2019-06-18

**Authors:** Gorik Ooms, Krista Kruja

**Affiliations:** 1 London School of Hygiene and Tropical Medicine, Department of Global Health and Development, 15-17 Tavistock Place, London, WC1H 9SH UK; 2 Independent Consultant, London, UK

**Keywords:** Global HIV/AIDS response, Universal health coverage, Integration

## Abstract

**Background:**

The international community’s health focus is shifting from achieving disease-specific targets towards aiming for universal health coverage. Integrating the global HIV/AIDS response into universal health coverage may be inevitable to secure its achievements in the long run, and for expanding these achievements beyond addressing a single disease. However, this integration comes at a time when international financial support for the global HIV/AIDS response is declining, while political support for universal health coverage is not translated into financial support. To assess the risks, challenges and opportunities of the integration of the global HIV/AIDS response into national universal health coverage plans, we carried out assessments in Indonesia, Kenya, Uganda and Ukraine, based on key informant interviews with civil society, policy-makers and development partners, as well as on a review of grey and academic literature.

**Results:**

In the absence of international financial support, governments are turning towards national health insurance schemes to finance universal health coverage, making access to healthcare contingent on regular financial contributions. It is not clear how AIDS treatment will be fit in. While the global HIV/AIDS response accords special attention to exclusion due to sexual orientation and gender identity, sex work or drug use, efforts to achieve universal health coverage focus on exclusion due to poverty, gender and geographical inequalities. Policies aiming for universal health coverage try to include private healthcare providers in the health system, which could create a sustainable framework for civil society organisations providing HIV/AIDS-related services. While the global HIV/AIDS response insisted on the inclusion of civil society in decision-making policies, that is not (yet) the case for policies aiming for universal health coverage.

**Discussion:**

While there are many obstacles to successful integration of the global HIV/AIDS response into universal health coverage policies, integration seems inevitable and is happening. Successful integration will require expanding the principle of ‘shared responsibility’ which emerged with the global HIV/AIDS response to universal health coverage, rather than relying solely on domestic efforts for universal health coverage. The preference for national health insurance as the best way to achieve universal health coverage should be reconsidered. An alliance between HIV/AIDS advocates and proponents of universal health coverage requires mutual condemnation of discrimination based on sexual orientation and gender identity, sex work or drug use, as well as addressing of exclusion based on poverty and other factors. The fulfilment of the promise to include civil society in decision-making processes about universal health coverage is long overdue.

## Background

The global response to HIV/AIDS stands as an unprecedented example of shared political and financial commitment by the international community. It has achieved significant results in removing the poverty barriers to accessing treatment, reaching key populations and other marginalised groups, involving communities in service provision and decision-making processes, and putting the right to health at its core [1].

In recent years, the international community’s health focus has started to shift from achieving disease-specific targets towards reaching the goal of universal health coverage (UHC) [2], which is prominently featured in the Sustainable Development Goals (SDGs) adopted in 2015 [3]. The World Health Organization (WHO) defines UHC as a situation in which “all people and communities can use the promotive, preventive, curative, rehabilitative and palliative health services they need, of sufficient quality to be effective, while also ensuring that the use of these services does not expose the user to financial hardship.” [4] Therefore, HIV/AIDS prevention and treatment should be included in UHC.

Global political support for UHC comes at a time when international funding for the global HIV/AIDS response is declining, while international funding specifically for UHC is stagnating [5]. (Due to its broad definition, all international funding for health can be considered as funding for UHC. However, we refer to funding intended to finance all elements of UHC in alignment with people’s needs, which is captured by the Institute of Health Metrics and Evaluation’s qualifications as sector-wide approach (SWAP) or health systems strengthening (HSS).) The simultaneous occurrence of global political support for UHC and declining international funding for the global HIV/AIDS response is probably not a coincidence. The international community suffers from HIV/AIDS “fatigue” [6]; increasing domestic resources for a form of UHC that includes HIV/AIDS and treatment would decrease the need of international funding for the global AIDS response. Thus, governments of low- and middle-income countries are expected to boost their domestic efforts to fund their national HIV/AIDS response and achieve UHC simultaneously.

From a right to health perspective, which acknowledges that all people have a right to access the health services they need, UHC should indeed be embraced as a global goal in lieu of disease-specific efforts. The global HIV/AIDS response should move “beyond the silos” in recognition of people’s complex health needs that require multiple health service responses [7]. Furthermore, the integration of the global HIV/AIDS response into national UHC efforts may be the only way to sustain its achievements. However, integration may also come at a substantial cost if domestic political will and resources are insufficient to achieve a truly inclusive and comprehensive form of UHC. Until countries are able and willing to fund their own HIV/AIDS treatment and key population programmes, within a context of UHC and the cost-effectiveness considerations and trade-offs that come with it, credible financial commitment must accompany the international community’s political commitment to UHC in order to ensure that progress made through the global HIV/AIDS response is maintained and expanded upon, rather than abandoned.

To assess the risks and opportunities of the integration of the global HIV/AIDS response into national UHC plans, we carried out assessments in Indonesia, Kenya, Uganda and Ukraine. These countries were selected because they are focus countries of the Partnership to Inspire, Transform and Connect the HIV response (PITCH) [8], which funded our study.

## Methods

Our country assessments were guided by the framework for the integration of targeted health interventions into health systems, as developed by Atun et al. [9] This framework is “intended to facilitate analysis in evaluative and formative studies of — and policies on — integration, for use in systematically comparing and contrasting health interventions in a country or in different settings to generate meaningful evidence to inform policy.” [9] We aimed to verify, in four countries, whether some of the alleged strengths of the HIV/AIDS response are indeed present in the ‘intervention’ (the HIV/AIDS response as it currently stands), and to what extent they are also present, or not, in the ‘adoption system’ (the UHC framework as it currently stands, or as it is being developed). Our aim was to perform a rapid assessment of the obstacles to integration, the risks of integration, and the opportunities which integration might offer.

Because of time constraints, we decided to focus on a limited number of alleged strengths of the global HIV/AIDS response. These strengths or achievements were selected – in slightly reformulated form – from the initial findings of the Lancet Commission on Defeating AIDS – Advancing Global Health [10], during a meeting with PITCH country teams in Amsterdam in January 2018. The Lancet Commission identified the following strengths or achievements:

- Activism and the leadership and engagement of civil society and people living with HIV;
- Multi-stakeholder partnerships and multi-sectoral collaboration;
- Political leadership;
- Human rights frameworks and instruments;
- Billions of dollars in financing;
- Global and local monitoring and accountability.

Our assessments focused on the following strengths or achievements:

- Matching political commitment with the mobilisation of financial resources (thus overcoming exclusion due to financial barriers);
- Efforts to identify and overcome stigmatisation and discrimination (thus overcoming exclusion due to societal barriers);
- Involvement of civil society in the provision of health services;
- Inclusion of civil society in essential decision-making processes about health services.

We carried out desk reviews and key informant interviews with representatives from government, civil society, and development partners in each country to elicit their views on the main risks and opportunities of integrating the HIV/AIDS response into UHC.

## Results

### Matching political commitment with the mobilisation of financial resources (thus overcoming exclusion due to poverty barriers)

Until 2001, the main obstacle to a comprehensive HIV/AIDS response – including treatment – in low- and middle-income countries was poverty, at individual and national levels. Most people living with HIV (PLHIV) in low- and middle-income countries were unable to pay the price of antiretroviral medicines asked by patent-holding pharmaceutical companies; governments of these countries were unable to fund or subsidise antiretroviral treatment (ART) for everyone who needed it.

In 2001, the situation changed. HIV/AIDS activism pressured pharmaceutical companies to restrain their interpretation of international intellectual property law. This first led pharmaceutical companies to abandon the litigation they had started against the Government of South Africa, and then to the World Trade Organisation (WTO), by the adoption of the Doha Declaration on the TRIPS agreement and public health, which confirmed that government were allowed to issue compulsory licences for patent-protected medicines – and thus to buy much cheaper generic equivalents [11].

However, for many low- and middle-income countries, especially those with significant HIV prevalence, even generic equivalents of antiretroviral medicines were too expensive. The United Nations General Assembly Special Session on HIV/AIDS of June 2001 called for the creation of a “global HIV/AIDS and health fund” [12], which became the Global Fund to fight AIDS, Tuberculosis and Malaria (Global Fund) [11]. The financing of the global HIV/AIDS response “skyrocketed” [13]. International financing for the global HIV/AIDS response was US$1.2 billion in 2002, reached a peak of $8.6 billion in 2014, and stood at $8.1 billion in 2017 [14].

According to UNAIDS, $26 billion are needed by 2020 “to be on track towards the end of AIDS as a global public health threat by 2030”., In 2016, only $19 billion were allocated (about $8 billion from international sources and, $11 billion from national sources) [15], still leaving a significant funding gap. The funding gap for UHC is of an entirely different magnitude. According to Stenberg et al., “an additional $274 billion spending on health is needed per year by 2030 to make progress towards the SDG 3 targets” in low- and middle-income countries [16]. It is important to note that SDG 3 is broader than UHC only, so if there is a funding gap of $274 billion per year for SDG 3, it does not necessarily mean a funding gap of $274 billion per year for UHC. However, Stenberg et al. estimate that about 75% of all costs for SDG 3 are costs for health systems, with health workforce and infrastructure (including medical equipment) as the main cost drivers, which are needed for UHC.

To assess the extent to which mobilisation of financial resources for the global HIV/AIDS response has overcome individual and national poverty barriers, it seems appropriate to start by examining the percentage of PLHIV who do (or do not) receive ART. According to UNAIDS [17]:

- In Indonesia, 14% of PLHIV are “on treatment”;
- In Kenya, 75% of PLHIV are “on treatment”;
- In Uganda, 72% of PLHIV are “on treatment”;
- In Ukraine, 40% of PLHIV are “on treatment”.

As Table 1 illustrates, there seems to be an inverse correlation between these countries’ gross domestic product (GDP) per capita and access to ART. A plausible explanation for this could be that poorer countries receive more international funding, which makes it easier to remove financial barriers. But there is also an inverse correlation between these countries’ HIV prevalence and access to ART. A plausible explanation for this correlation could be that in countries with a low adult HIV prevalence, the HIV/AIDS epidemic is concentrated among ‘key populations’, such as men having sex with men (MSM), sex workers (SW), and people who inject drugs (PWID). In this case, it would be likely that the main barrier to ART access is stigma and discrimination against key populations, rather than just financial barriers.

**Table 1:** PLHIV “on treatment”, GDP per capita and HIV prevalence among adults

	PLHIV “on treatment”	GDP per capita	HIV prevalence among adults (15–49 years)
Indonesia	14%	$3570	0.2%
Kenya	75%	$1595	4.8%
Uganda	72%	$582	5.9%
Ukraine	40%	$2640	0.9%

Sources: UNAIDS [17], World Bank [18], UNAIDS [19]

We found reports of financial barriers to ART at the individual level – people needing ART but not receiving it because they cannot pay for related expenses – in three out of four countries we assessed. In Indonesia, ART itself is free, but patients are supposed to pay a modest registration fee and costs of hospitalisation when needed [20]. In Kenya, the costs of frequent travels towards hospitals or health centres and registration fees exclude the poorest people from ART [21]. In Uganda as well, transportation costs seem to create a key financial barrier [22]. In Ukraine, we did not find evidence of financial barriers at the individual level [23].

International financial assistance that came with the global HIV/AIDS response also ‘removed’ poverty barriers at the national level, at least to some extent. However, there still is a long way to go. The UNAIDS 90–90-90 strategy claims: “By 2020, 90% of all people living with HIV will know their HIV status”, “90% of all people with diagnosed HIV infection will receive sustained antiretroviral therapy”, “90% of all people receiving antiretroviral therapy will have viral suppression” [24]. To achieve these targets, Kenya and Uganda will have to increase ART coverage from 75 and 72% respectively to 81%. They may find international financial support for that, even in the context of decreasing international financial support, due to being lower-middle-income and low-income countries, respectively, with high HIV prevalence. For Indonesia and Ukraine, which have higher income statuses and are therefore unlikely to receive the same international financial support, the challenge may seem enormous – increasing ART coverage from 14 and 40% respectively to 81%. The low HIV prevalence among adults in these countries – 0.4 and 0.9% – means that achieving ART coverage targets would require relatively modest financial needs.

According to WHO’s Global Health Expenditure Database:

- In Indonesia, the domestic government expenditure on health is 1.40% of the country’s GDP [25];
- In Kenya, the domestic government expenditure on health is 1.65% of the country’s GDP [25];
- In Uganda, the domestic government expenditure on health is 1.02% of the country’s GDP [25];
- In Ukraine, the domestic government expenditure on health is 2.85% of the country’s GDP [25].

Thus, all four countries are far away from the recommended target of 5% of GPD [26]. Even if we would use the more modest target of 3% of GDP, only Ukraine comes close.

Table 2 illustrates current (2016) levels of domestic government health expenditure, and potential levels if the 3% of GDP or 5% of GDP targets were achieved.

**Table 2:** Domestic government health expenditure, real and potential

	Domestic government health expenditure per capita in US$	Domestic government health expenditure as % of GDP	Domestic government health expenditure per capita in US$ - if it was 3% of GDP	Domestic government health expenditure per capita in US$ - if it was 5% of GDP
Indonesia	49.90	1.40	107.11	178.51
Kenya	23.95	1.65	43.66	72.76
Uganda	6.23	1.02	18.30	30.50
Ukraine	59.88	2.85	62.97	104.94

Sources: WHO [25], authors’ calculations

These figures draw particular concern around whether the international community’s shift of health attention towards UHC comes with increasing financial resources for UHC. So far, national funding for UHC remains low. International funding for health and for UHC stagnates [6], and there does not seem to be a strong global movement to change that. While it would be naïve to expect the international community to cover the $274 billion per annum UHC financing gap mentioned above, giving up on the idea that the international community could and should co-finance UHC efforts alongside increasing domestic efforts may become a self-filling prophecy: if there is no demand for international support for UHC, it will not be offered. However, there seems to be a growing consensus that efforts to move towards UHC should *not* rely on international funding. In 2016 and 2017, WHO issued three ‘health financing guidance’ papers. The first mentions international assistance in passing, and the main message – albeit subtly – is that governments should not rely on it: “The growing international focus on increasing domestic fiscal capacity provides a conducive environment to advocate for improving government revenue generation where the current revenue to [gross domestic product] is relatively low.” [27] The second again refers to “an environment of decreasing external assistance” as a reason to integrate disease-specific efforts, but does not challenge that environment [28]. Only the third mentions the importance of improving the quality of international assistance to make it suitable for UHC: “In those countries where external aid flows are significant [ministries of health should] include measures to improve predictability, for example moving external flows “on budget”, whilst bearing in mind that domestic resources may be offset i.e. reallocated elsewhere, as a result [29].”

The principle of ‘shared responsibility’ is a cornerstone of the HIV and AIDS response [30], but it is not (yet) a cornerstone of UHC. That raises the question whether governments of low- and middle-income countries can mobilise and allocate enough financial resources to cover the ‘financial UHC gap’ of $274 billion [16]. The answer is negative, at least for low-income countries: according to estimates of Gaspar of the International Monetary Fund (IMF), in the best case scenario low-income countries should be expected to raise less than half of the financial resources required to meet the SDGs [31].

Where are low- and middle-income countries’ governments looking for funding for UHC? According to McIntyre et al., “[m]any [sub-Saharan Africa] countries have declared a preference for achieving this through contributory health insurance schemes, particularly for formal sector workers, with service entitlements tied to contributions [32].” Our assessments confirm this. The Government of Kenya adopted a policy “to expand voluntary, contributory health insurance as one of the strategies to achieve UHC” [33]. However, the Government of Kenya seems committed to increasing government funding for UHC at the same time [34]. In Uganda, UHC has been taken up as a national policy priority as part of the Health Sector Development Plan [35, 36]. Although Uganda’s plans for UHC are far from finalised, a proposed mechanisms for financing and delivering healthcare is a national health insurance scheme [37, 38]. This trend is not limited to Africa. In 2012, the Government of Indonesia declared that it would achieve UHC by 2019, through a merger and expansion of existing health insurance schemes, forming the *Jaminan Kesehatan Nasional* (JKN) scheme [39]. The Ministry of Health of Ukraine adopted a National Strategy on Health Reform in 2015, which foreshadowed the introduction of a national health insurance scheme: “It is imperative to create conducive environment for the development of health insurance in long term perspectives [40].” However, this idea was badly received because the Constitution of Ukraine promises free healthcare. In reality, out-of-pocket health expenditure is as high as government expenditure and has been institutionalised through ‘charities’ set up by hospitals and health centres, to which patients are expected to contribute in order to receive ‘free’ healthcare services [41], but adopting a formal health insurance scheme – in obvious contradiction with the Constitution – seems to have been ‘one bridge too far’ for the Parliament of Ukraine. Meanwhile, the implementation of the reform has started with the introduction of family medicine, which is free. It remains to be seen if a health insurance scheme will be used to finance specialised healthcare [42].

### Efforts to identify and overcome stigmatisation and discrimination (thus overcoming exclusion due to societal barriers)

AIDS (acquired immunodeficiency syndrome) disproportionately affects key populations (MSM, SW, and PWID). Before HIV (human immunodeficiency virus) had been identified as the real cause of AIDS [43], the epidemic’s early years “were marked by the stigmatization of those living with the virus” [44]. Continuing discrimination against key populations and PLHIV at least partly explains “the gap between what is possible – greatly reduced levels of infection and deaths – and the continuing global epidemic” [44].

Table 3 below (including data from different sources and years) illustrates how the HIV/AIDS epidemic continues to affect some groups more than others.

**Table 3:** HIV prevalence among adults and some key populations

	HIV prevalence among adults (15–49 years)	HIV prevalence among sex workers	HIV prevalence among gay men and other men having sex with men	HIV prevalence among people who inject drugs
Indonesia	0.2%	5.3%	25.8%	28.8%
Kenya	4.8%	29.3%	18.2%	n.a.
Uganda	5.9%	37.0%	13.0%	26.7%
Ukraine	0.9%	5.2%	7.5%	22.6%

Sources: UNAIDS [17], UNAIDS [19], AVERT [45–47]

The availability of data demonstrating HIV prevalence between these populations highlights the challenges that social exclusion poses for eradicating HIV/AIDS. Data for other diseases or health issues might be disaggregated by geographic area, gender, income or other socio-economic status, but is unlikely to show disease prevalence for populations who are particularly vulnerable – to the disease and to exclusion from healthcare – because of societal stigmatisation.

These data are collected as a result of complex efforts. Sex work is illegal in Kenya, Uganda, and Ukraine [17]. In Indonesia there are laws that the police uses to harass SW, such as laws prohibiting the facilitation of acts of obscenity, trade in women, and living on the earnings of a female sex worker [48]. Homosexuality is illegal in Kenya and Uganda [17]. In Indonesia, the Ulama Council, which is the umbrella organization of many Islamic groups, issued a *fatwa* against homosexuality, on the basis that “homosexuality, whether lesbian or gay, and sodomy is legally haram and a form of crime”, and called for the criminalisation of lesbian, gay, bisexual and transgender activities [49]. The use of drugs is illegal in all four countries. Therefore, gathering these data requires creating an environment of trust in which the people concerned are willing to discuss their ‘illegal’ sexual orientation or behaviour. These data are largely collected as the by-product of tireless work by civil society organisations (CSO) of and for key populations, trying to overcome the stigmatisation of and discrimination against MSM, SW, PWID and PLHIV.

International financial support – and at times international political pressure – enabled the creation and development of these CSOs. CSOs can vary in type and mandate. They can include faith-based organisations, community-based groups and advocacy networks. The global HIV/AIDS response supports and acknowledges the importance of the work of CSOs in advancing human rights, influencing decision-making and also directly providing health services. One of the recommendations of the 2017 review of the national health sector response to HIV in Indonesia by WHO promotes “collaborative engagement with and support from these key populations” to support the government’s efforts to curb the epidemic country-wide and scale up access to ART, as both HIV treatment and prevention [50]. UNAIDS reports that “in Ukraine, for instance, stigma and discrimination towards people living with HIV in medical facilities has dropped from 22% (2010) to 8% (2016)”, and that “[c]ommunity-based organizations have major roles to play in efforts to reduce stigma and discrimination towards key populations, especially people who inject drugs, sex workers, gay men and other men who have sex with men, migrants and prisoners [17].”

The global HIV/AIDS response has had the strength of identifying and addressing stigmatisation and discrimination, at least in part because of the necessity of reaching marginalised populations to curb the HIV epidemic. However, this teleological approach to overcoming stigmatisation may have induce a three-fold flaw: first, that it did not address stigmatisation and discrimination beyond what was strictly necessary to remove access to testing and treatment barriers; second, that it overlooked other causes of exclusion than those directly related to key populations and; third, that it may not be convincing to the proponents of UHC, who focus on other inequalities, for which stigmatisation is a less urgent problem. Therefore, these achievements of the global HIV/AIDS response may not survive their integration within UHC.

However, there are indications that the cooperation between the Government of Ukraine and CSOs representing key populations will endure, even when international financial support for these CSOs would come to an end [51]. In Kenya, the Ministry of Health and CSOs are trying to develop innovative reporting mechanism that strike a balance “between the government’s obligation to abide by laws and communities’ commitment and right to work with, support and protect members of criminalized groups [52].” These findings are encouraging, but they also emphasise the fragility of the achievements of the global HIV/AIDS response. In Uganda, many Ugandan actors criticised international interference with national democratic procedures when wealthier countries threatened to discontinue financial support if a law aggravating the criminalisation of homosexuality was passed. However, Strand argues that international criticism helped to “strengthen human rights defenders” campaigning against the bill and mobilised domestic opponents to the law outside of human rights circles [53]. OutRight Action International – a New York based organisation advocating for the human rights of lesbian, gay, bisexual, transgender, intersex and queer (LGBTIQ) people – indicates that out of 41 LGBTIQ organisations in Indonesia, only one has formally registered as such, even though homosexuality has not (yet) been criminalised [54]. Can we find the strength of ‘overcoming societal exclusion’ in international and national strategies and efforts to move towards UHC?

“Leave no one behind” is the main slogan associated with UHC [55]. Proponents of UHC have shown, time and again, a deep commitment to equity, and to overcoming inequalities to accessing health services due to wealth or poverty, gender, and geographical factors [56]. However, references to UHC efforts aimed at overcoming inequalities due to discrimination based on sexual orientation and gender identity (SOGI) are hard to find, documents about UHC by HIV/AIDS groups excepted. Furthermore, many HIV/AIDS activists have not forgotten the “saga of agenda item 6.3 of the Executive Board” of WHO: the agenda point on “improving the health and well-being of lesbian, gay, bisexual and transgender persons” was removed due to pressure from states that felt this was a political rather than a public health issue [57–59].

At the national level, in the four countries we assessed, we found many references to exclusion due to poverty, gender and geography in documents about UHC, but no reference to discrimination based on SOGI. In countries where certain communities are marginalised widely in society, and their activities are prohibited by law, integrating HIV/AIDS prevention and treatment services into the broader mandate of UHC risks excluding them from general health services as well as HIV/AIDS specific programming. To overcome this, the proponents of UHC and HIV/AIDS will have to work together, moving beyond a results-oriented approach to stigmatisation, discrimination and exclusion, towards addressing the root causes of these problems.

### Involvement of civil society in the provision of services

The roles of CSOs involved in the HIV/AIDS responses in Indonesia, Kenya, Uganda and Ukraine go far beyond the advocacy described in the previous point. They are involved in HIV prevention, including harm reduction and HIV testing – voluntary counselling and testing (VCT) and self-testing. CSOs are also involved in guiding PLHIV through the healthcare system and in reaching out to PLHIV on ART who are missing appointments with their physicians or nurses. Furthermore, in Indonesia, Kenya and Uganda, CSOs have also set up HIV clinics serving the general population and marginalised communities, independent from or only partially integrated within the national health system. A WHO report about “examples of innovation and good practice on HIV prevention, diagnosis, treatment and care” includes examples from CSOs in Indonesia, Kenya, Uganda and Ukraine [60]. Most of these CSOs rely on international financial assistance.

There are two main reasons why the provision of HIV/AIDS services relies heavily on CSOs. The first was discussed above: the global HIV/AIDS response used CSOs to set up clinics where key populations did not have to fear stigmatisation. The second relates to the relative weakness of health services provision systems in some countries: wherever existing health systems were deemed too weak to provide uninterrupted AIDS treatment with robust follow up, the global HIV/AIDS response created a ‘parallel’ health system. As Topp et al. write: “Vertical HIV services have helped fulfill the mandate of emergency scale-up set by the WHO 3x5 initiative to rapidly enroll large numbers of HIV-infected patients by permitting implementers to bypass existing public health systems, set up parallel logistical and service-delivery arrangements and concentrate on intensively training select staff [61].” We will let history judge whether this was a mistake (or not). The question at hand is whether this ‘parallel’ health systems – or some of their features – would or should survive the integration of the global HIV/AIDS response into UHC.

Before trying to answer these questions, we would argue that the word ‘parallel’ may be somewhat misleading. In Indonesia, Kenya, and Uganda, faith-based organisations are important providers of HIV/AIDS-related healthcare services, but also of general healthcare services [62, 63]. These faith-based organisations vary in scope, style and function. Most “are now becoming more integrated with their national health systems through alignment of priorities, contracts, and service-level agreements [62]”. Many receive international financial support and rely on donations from their local faith-based constituencies. In this way, there is already a ‘model’ for all CSOs involved in the HIV/AIDS responses in Indonesia, Kenya, and Uganda to integrate with the UHC framework. Furthermore, this integration of CSOs, including faith-based organisations, aligns with one of the explicit goals of the UHC agenda: the integration of private (non-governmental) healthcare providers [64]. So, at least technically, UHC should be able to accommodate this integration. However, this model of integration into UHC comes with some challenges, which will be elaborated in the discussion section.

The situation is different in Ukraine: most healthcare is – or was, until recently – provided by governmental institutions; there never was a ‘parallel’ system for HIV/AIDS-related healthcare. As Lekhan et al. explain, in a 2015 report: “In 1991, Ukraine inherited an extensive and highly centralized Semashko health system (a hierarchical, nationally controlled system the staff of which were state employees), which it was not possible to maintain throughout the economic downturn that followed independence. There has been considerable decentralization in the system since independence; however, in most other respects, the system remains largely unreformed [41].” More recently, some reforms have taken place, with the introduction of family medicine: private practitioners who receive a set payment from the Ministry of Health for every patient that registers with them [42]. This model facilitates the establishment of private medical practice by CSOs who are delivering health care, including those who cater to key populations.

When it comes to HIV prevention (including harm reduction), testing, and outreach services, Ukraine is working on an innovative institutional arrangement. One of the elements of the ongoing health sector reform is the creation of the Public Health Centre of (the Ministry of Health) of Ukraine [40]. The Public Health Centre is mentioned in the progress reports of the European Union, Luxembourg and WHO partnership for UHC [65, 66]. It grew out of the Ukrainian Center for Combating AIDS, which became the Ukrainian Center for Socially Dangerous Disease Control, then the Public Health Centre of Ukraine. The (governmental) Public Health Centre is a principal recipient of the Global Fund, alongside the (non-governmental) Alliance for Public Health (originally the International HIV/AIDS Alliance in Ukraine). This may be because the Public Health Centre will gradually replace the Alliance for Public Health as principal recipient, while the Government of Ukraine will gradually replace the funding from the Global Fund with domestic funding [67–69]. This triple shift – from a non-governmental organisation which purchases preventive health services from CSOs to a governmental agency doing so, from international to national funding, and from an HIV/AIDS focus to a mandate for addressing various public health issues, including non-communicable diseases (NCDs) – will result in the Government of Ukraine ‘buying’ health promotion services from CSOs. This model presents a different kind of integration: an element of the HIV/AIDS response – collaboration with CSOs – that broadens its mandate and becomes a cornerstone of UHC.

### Inclusion of civil society in essential decision-making processes

The fourth and final strength or achievement of the global HIV/AIDS response we assessed is the involvement of CSOs in essential decision-making processes about health services. The WHO Global Program on AIDS – in many ways the precursor of UNAIDS – demonstrated a recognition of the importance of the inclusion of CSOs in the global HIV/AIDS response. The Global Program encouraged the formation of networks such as the Global Network of People Living With AIDS (GNP Plus), the International Council of AIDS Service Organisations (ICASO) and the International Community of Women Living with HIV/AIDS (ICW). When UNAIDS was launched as a joint programme of seven UN organisations, a Program Coordinating Board (PCB) was created to oversee its operations. The PCB includes representatives of CSOs, specifically PLHIV [70]. UNAIDS also encouraged and supported the creation of national AIDS commission or councils (NACs)– most of which include CSOs [71].

When the Global Fund was established, it embraced the principle of including CSOs in essential decision-making processes, and promoted this at the national level: to apply for funding, countries were – and still are – expected to establish a Country Coordination Mechanism (CCM), including CSOs [72]. In all four countries we assessed, key informants from CSOs confirm that NACs and CCMs are platforms where they can influence important decisions. Even if at times, CSO representatives indicate that they are mostly expected to endorse strategies already developed by the government with the ministry of health, CSOs representatives have a seat at the table and a meaningful voice: if they really disagree, they can. In Indonesia, however, the NAC has been dissolved [73].

The inclusion of civil society in HIV/AIDS-related decision-making processes we found in Indonesia, Kenya, Uganda and Ukraine, may fall short of the ideal of the 2018 Declaration of Astana - “We support the involvement of individuals, families, communities and civil society through their participation in the development and implementation of policies and plans that have an impact on health” [74] – if only because it is focused on one specific issue. Furthermore, while interviewees mentioned meaningful participation in local decision-making processes (provinces, counties, cities), which are becoming increasingly important, time constraints did not allow us to verify these responses. Nevertheless, the most important finding (or absence thereof) on this issue is a quasi-total absence of decision-making processes on UHC that include CSOs.

The global movement for UHC does not have the equivalent of UNAIDS or the Global Fund. It is establishing similar technical advisory and advocacy institutions, without a funding arm, however. In January 2004, WHO and the World Bank convened a first High-Level Forum on the Health Millennium Development Goals in Geneva in January 2004. A further two meetings of the High-Level Forum were held, in Abuja in December 2004 and a year later in Paris in November 2005. One of the conclusions was that WHO and the World Bank would “continue to collaborate in seeking new mechanisms to: influence the policy and practice of health development partners; improve technical support provided to countries to integrate health into poverty reduction strategies and accompanying budgets; and, explore new financing instruments for countries receiving limited donor support [75].” In September, 2007, the International Health Partnership (IHP) was launched, administered by WHO and the World Bank, and quickly evolved into the International Health Partnership and Related Initiatives (IHP+), absorbing other bilateral and multilateral initiatives [76].

In 2016, IHP+ transformed into UHC2030 [77]. The CSOs supporting the IHP+ became the Civil Society Engagement Mechanism (CSEM). Their original proposal included the creation of national groups, including a CSO national focal point “and CSO representatives from sectorial and sub-sectorial committees, including the Country Coordination Mechanism (CCM) of the Global Fund to fight AIDS, Tuberculosis and Malaria (GFATM) [78].”

Two years later, it is not clear if any CSEM national group actually exists and works. None of the CSO representatives we interviewed in Indonesia, Kenya, Ukraine or Uganda were aware of efforts to create a national CSEM group (although some had been invited to CSEM meetings and events in Geneva and elsewhere), at a time when crucial decisions about UHC are being taken. In Kenya, the Ministry of Health constituted a UHC Health Benefit Package Advisory Panel “for the design of an affordable, responsive health benefit package for the delivery of Universal Health Coverage”; this advisory panel includes the coordinator of the Health NGOs Network (HENNET) [79]. In Ukraine, some CSO representatives felt they are involved in the UHC process, although not nearly as meaningfully as they are in HIV and AIDS responses.

## Discussion

Before we enter into the discussion about strengths and opportunities of the integration of the global HIV/AIDS response into UHC, we should acknowledge the limitations of our study. Four countries make a small sample. In each country, we had only four to five working days for interviews. PITCH country teams helped us identify key informants, and the PITCH partnership is part of the global HIV/AIDS response; while all efforts were made to interview diverse stakeholders form government agencies, civil society, and development partners, the nature of identifying and contacting key informants may have introduced a bias.

However, we also want to emphasise what we believe to be a strength of our study: as far as we know, it is the first academic effort to systematically assess the risks and opportunities of the integration of the global HIV/AIDS response into UHC, which is quite surprising, given that the objective of integration of ‘vertical’ programmes is by no means new [2, 9]. We urge the readers to take our comments in this section with caution, and then to embark on further research.

Given our findings, what seem to be the main risks and opportunities of integration?

### Matching political commitment with the mobilisation of financial resources (thus overcoming exclusion due to poverty barriers)

The most imminent risk or perceived risk we identified is the possible return of exclusion from ART due to national and individual poverty. A combination of efforts to progress towards UHC relying on a national health insurance and integration of HIV/AIDS services into UHC may mean that continued access to ART becomes contingent on regular payment of health insurance contributions. This situation risks a decrease in treatment adherence by people on ART who prioritise other needs above health insurance payments when they feel healthy.

However, there are several ways to mitigate this risk, of which we will explore three here. First, there are other ways to progress towards UHC than national health insurance schemes. If countries decide to provide a package of ‘free’ healthcare, not contingent on health insurance contribution – i.e., financed by government revenue – ART could be included in that package, and thus remain free. Second, even if countries decide to adopt national health insurance as a key strategy to achieve UHC, they will keep some healthcare services outside of the package that is made contingent on regular health insurance fee payments, and ART could be one of these services. Third, even if countries decide to adopt national health insurance as a key strategy to achieve UHC, and decide to include ART in the package that is made contingent on regular health insurance fee payments, they will give waivers to some people, and could decide to give such waivers to all PLHIV.

While our assessments confirm what McIntyre et al. describe as a “declared preference” for achieving UHC via contributory health insurance schemes [32], not all countries of our assessment have taken a final irreversible decision on the issue. In Uganda and Ukraine, the introduction of a national health insurance is being considered, but the decision has not been taken yet: increasing government revenue to finance health services remains an option. In Kenya, the situation is not clear. On the one hand, a decision to use a national health insurance scheme to progress towards UHC has been taken [33]; but more recent media reports seems to suggest that healthcare at the primary level will remain free – and financed by government revenue – while the national health insurance would cover hospital care [34]. Only in Indonesia, the choice for the national health insurance route seems irreversible. Thinking through the potential consequences for PLHIV may help to reconsider ‘declared preferences’, and to consider – as McIntyre et al. indeed suggest – “how to increase domestic government revenue to fund quality health and other social services for all [32].”

Furthermore, in countries where governments decide to make progress towards UHC via a national contributary health insurance scheme, not all healthcare must be made contingent on regular insurance fee payments. Even a high-income country with a national health insurance coverage of 98.9%, like Belgium, makes exceptions for public health reasons: some vaccines are provided free to school children, regardless of whether parents have health insurance or not [80, 81]. The public health rationale of the UNAIDS’ 90–90-90 strategy provides a strong argument for offering ART even to those who cannot regularly contribute to the national health insurance scheme [24].

Finally, in countries where governments decide to try and achieve UHC via a national contributary health insurance scheme and to make ART contingent on enrolment in the national health insurance scheme, a waiver for all people needing chronic healthcare should be considered. All national health insurance schemes have mechanisms to avoid the exclusion of people who are unable to contribute. Usually, the allocation of free insurance cards is left to local authorities and communities, based on assessment of household income [82]. However, considering that even now (when and where ART is provided for free) transportation costs exclude the poorest people [21, 22], a general waiver for all people needing chronic care, decided by healthcare providers, should be considered.

While these options may appear to be technical, the choice between them will depend on public health policy and perhaps even political considerations. Scarce budgets in countries working towards UHC necessitate trade-offs to be made [88, 89]. Until now, HIV/AIDS programming has benefitted from policy choices made at the international level: the creation of the Global Fund and PEPFAR made HIV/AIDS programming ‘cheap’, at least from the perspective of countries receiving funding from PEPFAR and the Global Fund. Depending on how international funding for HIV/AIDS programming evolves, the integration of these efforts into UHC will change the way these trade-offs present themselves at the national level. Considering the data represented in Table 2 above, it should not be taken for granted that countries will continue to prioritise HIV/AIDS programming as they currently do: some may prioritise health or healthcare efforts that appear more cost-effective. This will depend on how they calculate or estimate the impact of ART on HIV transmission (or not). If they endorse the public health rationale of the UNAIDS’ 90–90-90 strategy, then the benefits of providing a year of ART should include the life years saved of a number people who will not become HIV positive – which could then justify keeping ART out of the package that is made contingent on regular health insurance contributions, or granting a waiver to all PLHIV. However, if countries do not endorse this rationale and take only the life years saved of people receiving ART into consideration, they could conclude that ART is not a priority, or that only the cheapest first line regimen should be subsidised.

Furthermore, besides financial consideration, the existence and persistence of discrimination against PLHIV and key populations must be considered and addressed when reflecting on technical solutions. In Indonesia, people must enrol in the health insurance scheme per family – not individually – and therefore they must go to the village or district where they are registered and present their ‘family card’ (*kartu keluarga*): all people mentioned on that card are enrolled together [83, 84]. Many members of key populations have been rejected by their families and live far away from the place where they are formally registered: they simply cannot enrol, even if they can afford and want to enrol.

When it comes to overcoming exclusion due to poverty barriers, the integration of the global HIV/AIDS response comes with opportunities as well. We already mentioned a first opportunity above: the potential consequences of including HIV/AIDS-related services into a package that is made continent on regular health insurance payments may tilt the balance towards government revenue funded healthcare services, which would avoid exclusion in many other healthcare areas. However, this would require a substantial increase of government revenue and a substantial increase of government revenue allocation to the health sector, which should not be taken for granted. Equitable financing requires a distributive effect: the wealthier subsidising the poorer, the healthier subsidising the less healthy people. For this to happen, pressure is required from the people who stand to benefit from the re-distributive effect. Effective alliances between HIV/AIDS advocates and other health advocates can be influential to increasing domestic financial resources for UHC. At the same time, as long as international funding streams for UHC remain a wishful thought, international funding streams for HIV/AIDS programming should not be abandoned, even though they may stand in the way of complete administrative, financial and operational integration [85]. Giving up on international financing for the HIV/AIDS response will not only inhibit the HIV/AIDS response, but it would also stunt progress towards UHC. In recognition of the importance of addressing multiple health needs, and drawing on international financing, HIV/AIDS and UHC advocates should work together to increase, improve and ‘de-verticalize’ international financial support for health.

Closing the financial UHC gap will require a significant increase in international and domestic funding. Though we have seen a peak for international funding for health in 2010 [5], it is not unreasonable to argue that international funding for UHC could and should increase. In 1970, a 0.7% of GDP target was set for countries to contribute as official development assistance [86]. This target has been re-agreed several times since 1970, with the 15 members of the European Union in 2004 agreeing to reach the target by 2015. The UK was one of the latest countries to reach the target 43 years after it was initially set, in 2013, along with Denmark, Luxembourg, Norway, Sweden and Germany, who has since dropped just below the target [87], Given these targets, and uneven progress towards them even decades later, it is not unreasonable that other high-income countries could and should increase international funding for UHC.

### Efforts to identify and overcome stigmatisation and discrimination (thus overcoming exclusion due to societal barriers)

A major risk of the integration of the global HIV/AIDS response into UHC is possible lost progress towards overcoming stigmatisation and discrimination of key populations. As we argued above, advances towards inclusivity of key populations is fragile. Though it is crucial to advances in the HIV/AIDS response, UHC primarily focuses on exclusion due to poverty, gender, and geographical factors. For example, the proposal for a CSEM mentions the importance of “systematic attention to the needs of the most marginalised and vulnerable populations so that no one is left behind”, but fails to mention any specific group [78].

While it is but natural that HIV/AIDS advocates and UHC advocates each focus on areas of exclusion that stand in the way of achieving their objectives most, it may hamper an effective alliance between them. UHC advocates and HIV/AIDS advocates share the same values. An alliance between proponents of UHC and proponents of the HIV/AIDS response would require increased efforts from proponents of UHC to condemning discrimination against PLHIV and key populations, and increased advocacy from proponents of the HIV/AIDS response for expanding access for all people to services for all health needs. Such an alliance would be very powerful and bring great opportunities. However, in our opinion, it would be a mistake to think that such an alliance can be built without international financial support and international political pressure. Scarce budgets in countries working towards UHC necessitate trade-offs to be made on which services and communities should be prioritised, and consequently who should not be prioritised to receive access or financial protection [88, 89]: people excluded by society are likely to be excluded from society’s health system too. As the Office of the Inspector General of the Global Fund understands, countries’ “ability to pay does not necessarily translate into a willingness to prioritize investments in the three diseases or support key populations affected by those diseases [90]”. The same is true for UHC: countries’ ability to finance UHC equitably does not imply that they will, nor does it imply that they will not exclude migrants, ethnic or religious minorities, or other groups. Increasing the scarcity of resources by reducing international funding for health overall may result in pitting HIV/AIDS advocates, UHC advocates, and advocates for other health issues, against each other.

### Involvement of civil society in the provision of services

The greatest opportunity coming with the integration of the global HIV/AIDS response into UHC could be the integration of services provided by CSOs within national health systems, if only because “[t]he goal of universal health coverage, as outlined in the Sustainable Development Goals, provides a renewed focus on the need to take a system perspective in designing policies to manage the private sector [64].” CSOs are technically or legally part of the private sector – even if they are not in spirit. This creates opportunities for CSOs providing HIV/AIDS-related services. However, we should not assume that the policies or arrangements that work for faith-based healthcare providers [62], will work for all CSOs that provide HIV/AIDS-related services. First, the financial contribution from ministries of health to faith-based healthcare providers is typically for healthcare services, not for prevention, outreach and other crucial services. Second, many faith-based healthcare providers charge out-of-pocket contributions, which they can because they are perceived as providing better quality healthcare services than public providers. Many CSOs providing HIV/AIDS-related services do not operate in this way. Third, most faith-based healthcare providers receive donations from their (faith-based) constituencies. This may not be possible for most CSOs providing HIV/AIDS-related services.

Ongoing efforts to secure the continuity of HIV/AIDS-related services when international funding is being reduced may also enrich UHC policies. The Public Health Centre of Ukraine, discussed above, could be a useful example to other countries [67, 68]. In Indonesia, the World Bank seems willing to support a similar effort, using the Indonesian National Development Planning Agency (*Badan Perencanaan Pembangunan Nasional* or *BAPPENAS*) as the contracting agency [91]. The Kenyan Ministry of Health’s attempt to work with CSOs seems very difficult, as it aims to develop reporting mechanisms that strike a balance “between the government’s obligation to abide by laws and communities’ commitment and right to work with, support and protect members of criminalized groups [52]”. In our opinion, such collaboration should lead to reconsideration of discriminatory laws rather than attempts to work around them.

While the inclusion of private providers is often associated with national health insurance schemes, there are other options. Contracting CSOs to deliver services which are financed by public resources requires a so-called ‘purchaser-provider split’, which can also be done by countries that opt for tax revenue financed health services [92].

### Inclusion of civil society in essential decision-making processes

The most urgent risk to be avoided, coming with the integration of the global HIV/AIDS response into UHC, could be the side-lining of CSOs from decision-making processes. In Indonesia, the NAC has already been dissolved, and the key informants we interviewed are very sceptical about the possibility of the CCM continuing if and when Global Fund financing will be discontinued.

While the goal of including civil society in UHC decision-making, as they were included in decision-making for HIV/AIDS, seems to exist at a global level, the current delay in doing so nationally poses a great risk. This could, in part, relate to the limited international financing accompanying domestic UHC efforts, unlike the significant HIV/AIDS international financing which was dependent on inclusion of civil society in decision-making mechanisms. The UCH2030 proposal to create national chapters of the CSEM should be implemented without further delay [78].

The achievements of the global HIV/AIDS response in this area are closely related to the international funding streams. Establishing a CCM, and working with the CCM, was (and still is) a condition for countries to have access to Global Fund funding. We suspect that not many countries would have established a CCM if that condition had not existed. Therefore, our finding that no national chapter of the CSEM exists to date should not be understood as a critique of the UHC movement. We acknowledge that without a global financing organisation for UHC demanding a country level CSEM, many governments and ministries of health may not be keen to establish a CSEM. But this is, in our opinion, yet another opportunity for an alliance between HIV/AIDS and UHC advocates: CCMs can be used as CSEMs for UHC. In a similar way as the Global Fund, but less conditional, the Global Financing Facility in Support of Every Woman Every Child (GFF) encourages CSO involvement in decision-making processes. The GFF is a relatively new – launched in 2015 – international funding mechanism, to help end preventable maternal and child deaths and improve the quality of life and health of women, children, and adolescents. In Kenya, it is the Health NGOs Network (HENNET) that coordinates CSO involvement in the GFF [93]; this is the same organisation that was included in UHC Health Benefit Package Advisory Panel [79]. Rather than waiting for a global funding mechanism for UHC that imposes the creation of a country level CSEM, CSOs could already work together and then claim their seat at the table.

## Conclusion

Integration presents opportunities for transferring successful components from the HIV/AIDS response to the UHC agenda, particularly the integration of CSO-provided services and engagement of CSOs in decision-making and advocacy. This engagement could lead to innovative health service delivery models, can further human rights advances and expand access to health services to all people regardless of their health needs. However, the existing progress made by the HIV/AIDS movement in reducing discrimination of marginalised populations, particularly based on SOGI, is in a fragile state. If integration of the global HIV/AIDS response into UHC is not accompanied by explicit condemnation of discrimination based on SOGI, there is potential for the key populations to be excluded from the right to health that UHC promises.

International financial and political support is required to prevent this retrogression and to ensure that financial resources for achieving UHC and HIV/AIDS goals are sufficient. In limited resource settings, it would be all too easy for politically unpopular groups to be excluded when resource-allocation decisions are made. This poses a risk of resurgence of the HIV/AIDS epidemic and a loss of the decades of progress so far.

While this paper outlines several key risks and opportunities for integrating the global HIV/AIDS response into UHC, more research is urgently needed to accompany and guide the ongoing process of integration. We would highlight the following two areas for future research:

- We need a better understanding of the potential costs and benefits, in the short and long run, of the different technical options of integrating HIV/AIDS financing into the financing of other healthcare services, including the long-term impact of short-term consequences of reduced ART adherence;
- We also need a better understanding of the landscape in which CSOs operate, as advocates and as services providers, at national and local levels, how they could build alliances that have not yet emerged, and why such alliances have not yet emerged.

We also think that this research should be undertaken in more countries.

In conclusion, we would like to respond to Poku’s comment, according to which the integration of the global HIV/AIDS response into UHC is “a case of ‘the right thing at the wrong time’” [94], with a rhetorical question. Will there ever be a perfect time, or even a good time for this integration? It is happening.

## Data Availability

The research is based on findings from the grey and academic literature as well as primary data collected via key informant interviews in each country. Due to the sensitive nature of the topic, recordings of the interviews will not be shared.

## Abbreviations

AIDS: Acquired immunodeficiency syndrome
ART: Antiretroviral treatment
CCM: Country coordination mechanism
CSEM: Civil society engagement mechanism
CSO: Civil society organisation
GDP: Gross domestic product
Global Fund: Global Fund to Fight AIDS, Tuberculosis and Malaria
GNP Plus: Global Network of People Living With AIDS
GRID: Gay related immunodeficiency
HENNNET: Health NGOs Network
HIV: Human immunodeficiency virus
HSS: Health systems strengthening
ICASO: International Council of AIDS Service Organisations
ICW: International Community of Women Living with HIV/AIDS
IHP: International Health Partnership
IHP+: International Health Partnership plus related initiatives.
IMF: International Monetary Fund
LGBTIQ: Lesbian, gay, bisexual, transgender, intersex and queer
MDGs: Millennium Development Goals
MSM: Men who have sex with men
NAC: National AIDS commission/council
NCD: Non-communicable diseases
PCB: Program Coordination Board (of UNAIDS)
PITCH: Partnership to Inspire, Transform and Connect the HIV response
PLHIV: People living with HIV
PWID: People who inject drugs
SDGs: Sustainable Development Goals
SOGI: Sexual orientation and gender identity
SW: Sew workers
SWAP: Sector-wide approach
UHC: Universal health coverage
UHC2030: *“the global movement to build stronger health systems for UHC”*
VCT: Voluntary counselling and testing
WHO: World Health Organisation
